# Whole Genome Sequence Analysis of CTX-M-15 Producing *Klebsiella* Isolates Allowed Dissecting a Polyclonal Outbreak Scenario

**DOI:** 10.3389/fmicb.2018.00322

**Published:** 2018-02-23

**Authors:** Laura Becker, Stephan Fuchs, Yvonne Pfeifer, Torsten Semmler, Tim Eckmanns, Gerit Korr, Dagmar Sissolak, Michael Friedrichs, Edith Zill, Mei-Lin Tung, Christian Dohle, Martin Kaase, Sören Gatermann, Holger Rüssmann, Matthias Steglich, Sebastian Haller, Guido Werner

**Affiliations:** ^1^Department of Infectious Diseases, Robert Koch Institute, Berlin, Germany; ^2^Postgraduate Training for Applied Epidemiology, Robert Koch Institute, Affiliated to the European Programme for Intervention Epidemiology Training, European Centre for Disease Prevention and Control (ECDC), Stockholm, Sweden; ^3^Department of Infection Control, Medical Disaster Control and Environmental Health Control, Department of Public Health, Berlin, Germany; ^4^MEDIAN Klinik Berlin-Kladow, Berlin, Germany; ^5^Medical Care Centre Labor 28 GmbH, Berlin, Germany; ^6^National Reference Centre for Multidrug-Resistant Gram-Negative Bacteria, Department for Medical Microbiology, Ruhr-University Bochum, Berlin, Germany; ^7^Immunology and Laboratory Medicine, Institute for Microbiology, HELIOS Klinikum Emil von Behring, Berlin, Germany; ^8^Leibniz Institute DSMZ, German Collection of Microorganisms and Cell Culture, Braunschweig, Germany

**Keywords:** ESBL, outbreak analysis, NGS, strain typing, CTX-M-15

## Abstract

Extended-spectrum β-lactamase (ESBL) producing *Klebsiella pneumoniae* pose an important threat of infection with increased morbidity and mortality, especially for immunocompromised patients. Here, we use the rise of multidrug-resistant *K. pneumoniae* in a German neurorehabilitation center from April 2015 to April 2016 to dissect the benefit of whole genome sequencing (WGS) for outbreak analyses. In total, 53 isolates were obtained from 52 patients and examined using WGS. Two independent analysis strategies (reference-based and -free) revealed the same distinct clusters of two CTX-M-15 producing *K. pneumoniae* clones (ST15, *n* = 31; ST405, *n* = 7) and one CTX-M-15 producing *Klebsiella quasipneumoniae* strain (ST414, *n* = 8). Additionally, we determined sequence variations associated with antimicrobial resistance phenotypes in single isolates expressing carbapenem and colistin resistance, respectively. For rapid detection of the major *K. pneumoniae* outbreak clone (ST15), a selective triplex PCR was deduced from WGS data of the major outbreak strain and *K. pneumoniae* genome data deposited in central databases. Moreover, we introduce two novel open-source applications supporting reference genome selection (*refRank*; https://gitlab.com/s.fuchs/refRank) and alignment-based SNP-filtering (*SNPfilter*; https://gitlab.com/s.fuchs/snpfilter) in NGS analyses.

## Introduction

Extended-spectrum β-lactamase (ESBL)-producing *Klebsiella pneumoniae* are important nosocomial pathogens (Gupta et al., 2003). In particular, immunocompromised persons are susceptible to severe infections caused by *K. pneumoniae* (Podschun and Ullmann, 1998). Nosocomial outbreaks with *K. pneumoniae* are frequently reported from (neonatal) intensive care units (Calbo and Garau, 2015; Stapleton et al., 2016). Within the last few years, the benefit of whole genome sequencing (WGS) to analyse, confirm, and understand better outbreak scenarios has been shown for distinct settings (Quainoo et al., 2017). For instance, three temporally separated case clusters in a Nepali hospital were found to be in fact caused by a single *K. pneumoniae* strain using WGS-based analysis strategies (Stoesser et al., 2014). Furthermore, WGS-based analysis of an ongoing detection of ESBL-producing *K. pneumoniae* revealed prevalence of the outbreak strain over a period of several months while extensive infection control measures have been performed. In addition, WGS analysis allowed identification of the origin of the outbreak years before the first clinical and supposed index case was notified (Haller et al., 2015). Nevertheless, most of these studies were performed retrospectively. Although real-time WGS analysis during nosocomial outbreaks is, in principle, a realistic scenario nowadays, limitations in terms of costs, quick access to relevant techniques and qualified personnel and other factors exist in practice. Deducing quick, simple but reliable and specific tests such as PCR-based assays using WGS data would be one of the possible solutions to existing restrictions.

Formerly, *K. pneumoniae* has been divided into three phylogroups (KpI, KpII, KpIII) and meanwhile these groups have been classified as distinct species (*K*. *pneumoniae, Klebsiella quasipneumoniae*, and *Klebsiella variicola*; Brisse and Verhoef, 2001; Brisse et al., 2004, 2014; Rosenblueth et al., 2004). Accordingly, the term *K. pneumoniae sensu stricto* refers to isolates belonging to KpI, while the term *K. pneumoniae sensu lato* comprises isolates of all three phylogroups/species. In previous studies, the majority of clinical *Klebsiella* isolates was found to belong to KpI (69–82%), while KpII and KpIII represent a remarkably smaller proportion (6–8 and 11–24%, respectively; Brisse et al., 2004; Maatallah et al., 2014). A similar composition was reported for ESBL-producing *Klebsiella* isolates from a Spanish hospital (Valverde et al., 2008). In contrast to standard diagnostic procedures (MALDI TOF MS) and phenotypic assays for species prediction, only genotypic approaches based on allelic sequences of *gyrA* and *parC* or chromosomal β-lactamase genes (*bla*) allow a reliable differentiation between these species (Brisse and Verhoef, 2001; Brisse et al., 2004). *K. pneumoniae* was shown to be associated with *bla*_SHV_, *K. quasipneumoniae* with *bla*_OKP_ and *K. variicola* with *bla*_LEN_ (Haeggman et al., 2004). This genotypic information could also be deduced from WGS data. In a recent report Long and colleagues used WGS of 1,777 clinical ESBL-producing *Klebsiella* isolates, all initially determined as *K. pneumoniae* by MALDI TOF MS, to further resolve their subspecies into 13 *K. variicola* and 15 *K. quasipneumoniae* (0.7 and 0.8%, respectively; Long et al., 2017). In addition, specific PCRs were developed for the identification of *K. pneumoniae sensu stricto* and *K. variicola*, respectively (Bialek-Davenet et al., 2014; Garza-Ramos et al., 2015). However, these methods are usually not part of the standard diagnostic routine in clinical laboratories.

From April 2015 onwards, an increased incidence of patients colonized or infected with ESBL-producing *K. pneumoniae* was observed in a neurorehabilitation center in Germany. Here, we report the WGS-based analysis of isolates from this setting including deduction of a selective PCR screening for rapid detection of the major *K. pneumoniae* clone and relevant resistance characteristics. Furthermore, we introduce *refRank* and *SNPfilter*, two bioinformatics' tools developed for mapping-based approaches to identify the best matching reference genome and to filter single nucleotide polymorphisms (SNPs) in sequence alignments, respectively.

## Materials and methods

### Collection of isolates

Isolation of bacteria and primary diagnostics including biochemical and automated species identification via MALDI-TOF MS (MALDI Biotyper, Bruker Daltonik, Bremen, Germany) as well as antibiotic susceptibility testing (minimum inhibitory concentration determined using BD Phoenix System) were performed by the routine diagnostic laboratory of the hospital. ESBL-positive isolates were identified using Chromagar ESBL (Mast Group, Reinfeld, Germany). A total of 53 bacterial isolates (52 patients) collected between April 2015 and April 2016 were sent to the Robert Koch Institute, Germany and the National Reference Centre for multidrug-resistant gram-negative bacteria for molecular typing (Table 1).

**Table 1:** Overview of characteristics of all *Klebsiella* isolates from the neurohabilitation centre, April 2015–April 2016.

**No**	**ID^*a*^**	**Isolation date^*b*^**	**Material^*c*^**	**MLST^*d*^**	**wzi^*e*^**	**Cluster WGS^*f*^**	**PFGE type^*g*^**	**Multiplex PCR^*h*^**	**Alterations in porin genes^***i***^**		**β-lactamase genes^***j***^**				
									***ompK35***	***ompK36***	***bla*_TEM_**	***bla*_CTX−M_**	***bla*_SHV_**	***bla*_OXA_**	***bla*_OKP−B_**
1	669/15	16 April 2015	Urinary catheter	ST15	93	1	n.d.	+	+	+	–	15	28	–	–
2	652/15	19 April 2015	Tracheal secretion	ST15	93	1	n.d.	+	+	+	1B	15	28	534	–
3	656/15	24 April 2015	Tracheal secretion	ST37	83	–	n.d.	–	+	+	–	14	11	–	–
4	665/15	8 May 2015	Tracheal secretion	ST15	93	1	n.d.	+	+	+	1B	15	28	534	–
5	666/15	8 May 2015	Tracheal secretion	ST15	93	1	1a	+	+	+	–	15	28	534	–
6	667/15	3 June 2015	Urinary catheter	ST15	93	1	1c	+	+	+	1B	15	28	534	–
7	657/15	6 June 2015	Nasal swab	ST15	93	1	n.d.	+	+	+	1B	15	28	534	–
8	661/15	10 June 2015	Nasal swab	ST15	93	1	n.d.	+	+	+	1B	15	28	534	–
9	659/15	15 June 2015	Urinary catheter	ST15	93	1	n.d.	+	+	+	1B	15	28	534	–
10	653/15	18 June 2015	Throat swab	ST101	137	–	n.d.	–	Stop (C184-)	+	1A	15	1	1; 9-like	–
11	654/15	23 June 2015	Urinary catheter	ST15	93	1	n.d.	+	+	+	1B	15	28	534	–
12	380/16	29 June 2015	Tracheal secretion	ST15	93	1	1a	+	+	+	1B	15	28	534	–
13	379/16	29 June 2015	Tracheal secretion	ST15	93	1	1a	+	+	+	1B	15	28	534	–
14	672/15	2 July 2015	Tracheal secretion	ST15	93	1	1a	+	+	+	1B	15	28	534	–
15	377/16	9 July 2015	Tracheal secretion	ST15	93	1	n.d.	+	+	+	1B	15	28	534	–
16	658/15	9 July 2015	Tracheal secretion	ST15	93	1	n.d.	+	+	+	1B	15	28	534	–
17	378/16	16 July 2015	Tracheal secretion	ST2382	280	–	5a	–	+	Stop (−227C and −228C)	–	1	196	–	–
18	668/15	24 July 2015	Urinary catheter	ST15	93	1	n.d.	+	+	+	1B	15	28	534	–
19	655/15	29 July 2015	Urinary catheter	ST15	93	1	n.d.	+	+	+	1B	15	28	534	–
20	662/15	19 Aug. 2015	Tracheal secretion	ST15	93	1	1b	+	+	+	1B	15	28	534	–
21	670/15	28 Aug. 2015	Urinary catheter	ST48	62	–	4b	–	+	+	1B	15; 27	1	1	–
22	660/15	1 Sep. 2015	Rectal swab	ST15	93	1	1a	+	+	+	1B	15	28	534	–
23	675/15	8 Sep. 2015	Rectal swab	ST15	93	1	1a	+	+	+	1B	15	28	534	–
24	671/15	14 Sep. 2015	Urinary catheter	ST15	93	1	1a	+	+	+	–	15	28	–	–
25	676/15	14 Sep. 2015	Rectal swab	ST414	44	3	3a	–	+	+	–	15	–	–	8
26	673/15	15 Sep. 2015	Tracheal secretion	ST15	93	1	1a	+	+	+	1B	15	28	534	–
27	674/15	15 Sep. 2015	Urine	ST414	44	3	3a	–	+	+	–	15	–	–	8
28	677/15	23 Sep. 2015	Rectal swab	ST15	93	1	1d	+	+	+	1B	15	28	534	–
29	678/15	23 Sep. 2015	Rectal swab	ST15	93	1	1a	+	+	+	–	15	28	534	–
30	679/15	29 Sep. 2015	Rectal swab	ST15	93	1	1a	+	+	+	1B	15	28	534	–
31	680/15^*k*^	29 Sep. 2015	Rectal swab	ST15	93	1	1a	+	+	+	1B	15	28	534	–
32	682/15	29 Sep. 2015	Rectal swab	ST15	93	1	1a	+	+	+	1B	15	28	534	–
33	681/15	29 Sep. 2015	Rectal swab	ST414	44	3	3a	–	+	+	–	15	–	–	8
34	684/15^*k*^	29 Sep. 2015	Tracheal secretion	ST15	93	1	1a	+ ^*^	IS	Stop (C194-)	1B	15	28	–	–
35	683/15	6 Oct. 2015	Tracheal secretion	ST15	93	1	1a	+	+	+	1B	15	28	534	–
36	663/15	28 Oct. 2015	Rectal swab	ST15	93	1	1a	+	+	+	1B	15	28	534	–
37	54/16	27 Oct. 2015	Urine	ST15	93	1	1a	+	+	+	1B	15	28	534	–
38	55/16	7 Nov. 2015	Rectal swab	ST405	143	2	2a	–	Stop (G947A)	+	1B	15	76	–	–
39	56/16	7 Nov. 2015	Rectal swab	ST405	143	2	2a	–	+	+	1B	15	76	–	–
40	57/16	7 Nov. 2015	Rectal swab	ST405	143	2	2a	–	+	+	1B	15	76	–	–
41	58/16	10 Nov. 2015	Unknown	ST405	143	2	2a	–	+	+	1B	15	76	–	–
42	59/16	16 Dec. 2015	Urine	ST405	143	2	2a	–	+	+	1B	15	76	–	–
43	382/16	17 Dec. 2015	Urine	ST15	24	–	6a	–	+	+	–	15	28	1	–
44	383/16	12 Jan. 2016	Throat swab	ST29	85	–	7a	–	+	+	1B	15	83	1	–
45	381/16	30 Jan. 2016	Rectal swab	ST405	143	2	2a	–	+	+	1B	15	76	–	–
46	384/16	9 Feb. 2016	Rectal swab	ST15	93	1	1e/f	+	+	+	1B	15	28	534	–
47	385/16	8 Mar. 2016	Rectal swab	ST405	143	2	2a	–	+	+	1B	15	76	–	–
48	386/16	14 Mar. 2016	Rectal swab	ST414	44	3	3a	–	+	+	–	15	–	–	8
49	388/16	6 Apr. 2016	Tracheal secretion	ST14	2	-	8a	–	+	+	1B	15	28	1	–
50	389/16	9 Apr. 2016	Rectal swab	ST414	44	3	3a	–	+	+	–	15	–	–	8
51	390/16	9 Apr. 2016	Rectal swab	ST414	44	3	3a	–	+	+	–	15	–	–	8
52	391/16	9 Apr. 2016	Rectal swab	ST414	44	3	3a	–	+	+	–	15	–	–	8
53	392/16	9 Apr. 2016	Rectal swab	ST414	44	3	3a	–	+	+	–	15	–	–	8

a *ID, laboratory strain number*.

b *Isolation date, time point of sample isolation*.

c *Material, sampling site*.

d *MLST, multilocus sequence type according to the Klebsiella Pasteur MLST definition (http://bigsdb.pasteur.fr/)*.

e *wzi, capsule type based on sequence analysis of the wzi gene*.

f *cluster WGS, cluster classification based on the WGS analysis*.

g *PFGE-type, classification based on PFGE analysis (Tenover et al., 1995), n.d., not determined*.

h multiplex PCR, result of the cluster 1 specific PCR (positive: all three bands present, positive;

* *, PCR product of 4160 kb primers is missing; negative, no PCR product or only amplification of the hem-F/-R product)*.

i *Alterations in porin genes, results of sequence analysis of the porin genes ompK35 and ompK36, +, intact gene; IS, disruption of the gene due to insertion of a transposase; stop, premature stop codon due to mutation(s) in the gene; in brackets, details on mutation*.

j *β-lactamase genes, β-lactamase gene alleles identified based on WGS data (using Resfinder plus manual completion)*.

k *Isolates 680/15 and 684/15 originated from the same patient*.

### Phenotypic resistance testing

Antibiotic susceptibilities were determined using the VITEK 2 system (card AST-N248, bioMérieux) and results were interpreted according to the EUCAST breakpoints (version 7.1). In addition, Etest (bioMérieux) for imipenem, ertapenem, meropenem, and colistin was used (Table S1). For a set of 12 out of 53 isolates representing all major clonal variants the MIC for colistin was determined using broth microdilution according to EUCAST.

### Whole genome sequencing

Bacteria were cultivated in Brain Heart Infusion (BHI) broth. DNA was extracted from overnight cultures using the MagAttract Kit (Qiagen, Hilden, Germany) and the DNeasy Blood & Tissue Kit (Qiagen) in line with the manufacturer's instructions. Qubit dsDNA HS Assay Kit (Invitrogen/Thermo Fisher Scientific, Karlsruhe, Germany) was used for DNA quantification. Sequencing libraries were prepared applying the Nextera XT Kit (Illumina, San Diego, USA) and sequenced on an Illumina Miseq using v3 chemistry (2 × 300 bp) according to the manufacturer's protocol.

### Mapping-based WGS data analysis

Raw reads were trimmed applying Trimmomatic (version 0.32, parameters: illuminaclip off, slidingwindow 4:15, leading 3, trailing 3, crop off, headcrop off, minlen 36, avgqual off; Bolger et al., 2014) and mapped to the reference genome of *K. pneumoniae* PMK-1 (NZ_CP008929) using BWA-SW (version 0.7.13-r1126, default parameters) (Li and Durbin, 2009). The reference genome was selected using *refRank* (version 1.0.0; see below). SNPs were called using Varscan v2.3 (Koboldt et al., 2012) and variant positions were filtered using *SNPfilter* (version 2.2.0; see below). Based on the aligned variant positions maximum-likelihood trees were calculated using RAxML (version 8.2.7, model GTR GAMMA, 100 bootstraps; Stamatakis, 2014). The genome sequence of *Klebsiella oxytoca* KONIH1 (accession: CP008788) was included to root the tree displaying the phylogenetic relationship of all sequenced isolates. For this purpose, artificial Illumina reads were generated from the FASTA-sequence downloaded from NCBI using ART (version 2.5.8, parameters: system MiSeq v3, read length 250, paired end, fragment length 600, standard deviation 300, coverage 100; Huang et al., 2012) and mapped to the reference genome as described above for the sequenced isolates.

### Reference genome selection using *refRank*

To optimize the selection of a reference sequence for NGS read mapping, we created a Python-based application called *refRank* which provides a coverage-based reference ranking (Figure S1). For this purpose, datasets of single or paired-end reads can be aligned against a collection of defined reference sequences using BWA-SW (Li and Durbin, 2009) or BWA-MEM [arXiv:1303.3997v2 (q-bio.GN)]. Computational costs can be reduced by using only a fraction of randomly picked (paired-end) reads of each dataset (e.g., 10% of all reads). Per base coverage is then determined using SAMtools (Li et al., 2009) and normalized to the reference length and the number of total (mapped and unmapped) reads according to formula (1).

(1) $$\begin{array}{r} C=\;\frac{\sum_{i=1}^{L}c_{i}}{L\times N} \end{array}$$

*L* is reference sequence length, *c*_*i*_ is coverage at base *i*, *N* is total read number.

Dataset-specific reference ranking is based on the calculated C scores. Additionally, a global reference ranking based on all datasets is provided by calculating the grand average of reference-specific C scores. The source code for *refRank* is freely available under the terms of the GNU General Public License v3.0 (https://gitlab.com/s.fuchs/refRank).

In the present study, *refRank* (version 1.0.0) was used with default parameters. The entire collection of completed genomic sequences of *K. pneumoniae* (*n* = 63) and *K. quasipneumoniae* (*n* = 1) available on RefSeq (accessed at 19th July 2016) was used as reference dataset. All 53 raw read datasets were trimmed using Trimmomatic (Bolger et al., 2014; see above). According to the reference ranking (not shown), the genomic sequence of *K. pneumoniae* PMK-1 (NZ_CP008929) has been selected as a reference for further analysis.

### Variant site filtering using *SNPfilter*

A python-based application called *SNPfilter* was developed to condense the alignment of all reconstructed sequences to variant positions only and, thus, to significantly reduce computational costs of subsequent phylogenetic analyses. Optionally, sites can be excluded based on (i) ambiguous base calls and/or deletions, (ii) SNP accumulation (based on exclusion distance), and (iii) user-defined regions (based on genomic coordinates). Importantly, circular replicon topologies can be considered when applying an exclusion distance for accumulated variant positions. The output of *SNPfilter* provides different files containing (i) aligned variant sites that meet the filter criteria (FASTA format), (ii) general information such as sequence names, number of sites containing ambiguous base calls or gaps, number of variants before and after filtering (TXT format), and (iii) coordinate-specific filter status and sequence information (CSV format). The source code for *SNPfilter* is freely available under the terms of the GNU General Public License v3.0 (https://gitlab.com/s.fuchs/snpFilter).

In the present study sites containing gaps or ambiguous base calls were excluded. To exclude SNPs in repetitive regions, the reference sequence (NZ_CP008929) was analyzed using the repeat analysis tool in Kodon (Applied Maths) version 3.62 PHASTER (Arndt et al., 2016). Additionally, SNPs in regions of annotated phages and transposases were rejected (not shown).

### Maximum common genome

Phylogenetic relationship was determined on the basis of the Maximum Common Genome (MCG) (von Mentzer et al., 2014), the set of orthologous genes that are present in all 54 genomes. A gene prediction was performed on the de novo assembled contigs by use of the Prokaryotic Dynamic Programming Genefinding Algorithm (Prodigal) (Hyatt et al., 2010). The obtained coding sequences where then subsequently clustered using USEARCH v7 (Edgar, 2010) with thresholds of 70% similarity on nucleotide level and 90% coverage to determine the set of orthologous genes (*n* = 1,117) of all genomes. This set was then used as a reference to extract the corresponding allelic variants of the MCG genes from the 54 genomes using PLAST v2.3.1 (Nguyen and Lavenier, 2009). Afterwards the alleles for each gene were aligned with MUSCLE v3.8.31 (Edgar, 2004) and concatenated.

The resulting alignment (1.161 Mbp in length) was used to infer a maximum likelihood phylogeny with RAxML version 8.1.14 using a General Time Reversible model and gamma correction for among site rate variation (Stamatakis, 2014).

### *De novo* assembly

Raw reads were trimmed applying Trimmomatic (version 0.32, parameters: Illuminaclip off, maxinfo 50:0.8, leading 3, trailing 3, crop off, headcrop off, minlen 36, avgqual off; Bolger et al., 2014). Trimmed reads were *de novo* assembled using A5-miseq (version 20150522) with default parameter (Coil et al., 2015).

### Cluster specific multiplex PCR

The multiplex PCR was designed to allow a rapid identification of isolates belonging to the major outbreak strain (cluster 1). To achieve sufficient discriminatory power three targets had to be considered: (i) a region specific for ST15, (ii) a signature specific for cluster 1 isolates, and (iii) a unique region within a plasmid present in cluster 1 isolates (Table 2). The first primer pair (hem-F/-R) amplifies a presumably ST15-specific region in the ST15 reference genome (NZ_CP008929), which was only covered by reads obtained from cluster 1 isolates (BWA-SW, default parameters). The respective primer pair binds upstream and inside of the hemolysin secretion/activation protein CDS (locus tag PMK1_RS11760) and BLAST search (web BLAST, nucleotide collection, March 2016) of the amplified region did not reveal any further hits in *K. pneumoniae* isolates (Altschul et al., 1990). Reads of isolate 652/15 that did not map onto the reference genome were *de novo* assembled in Geneious (version 9.1.6, Geneious assembler, Biomatters Ltd., 30% of data; Kearse et al., 2012). The second primer pair (unique-F/-R) was designed to amplify a region within a contig derived from *de novo* assembly exhibiting neither BLAST hits within the *Klebsiella* species nor in plasmid sequences. The third primer pair (4.160 kb-F/-R) was designed to amplify a region within a 4.160-kb plasmid (see section Results) without any BLAST hits. A number of unrelated *K. pneumoniae* isolates including three ST15 isolates of our institute's strain collection (10 isolates with ESBL production and 10 carbapenemase-producing isolates from diverse hospitals in Germany) was chosen to evaluate the specificity of the multiplex PCR (Table S2).

**Table 2:** Primer sequences of the cluster 1 specific multiplex PCR designed in this study.

**Primer name**	**Sequence (5′−3′)**	**Product size**
4.160 kb-F	GTTAGAATCAAGATGCACAGTACGC	606 bp
4.160 kb-R	CCATGGATTGAACTTGGTGTGAG	
hem-F	GGGTTTGGTTGTATTAAATGCCACG	379 bp
hem-R	CCCAATCGCTTTATTTTCCTGACG	
Unique-F	CACAAATTCCCATCTGAGGTCATG	265 bp
Unique-R	CCACCAAAGCTAAATACTTCGCTG	

### WGS-based conduction of MLST, *wzi* type, and resistance pattern

WGS data of all isolates were analyzed using Resfinder 2.1 (Zankari et al., 2012) and the MLST tool (Larsen et al., 2012) provided by the Centre for Genomic Epidemiology (https://cge.cbs.dtu.dk/services/). Presence of *mcr-1-5* genes were tested using ResFinder 3.0. Using Geneious (Biomatters Ltd.) the contigs derived from *de novo* assembly using A5-miseq were checked for alterations within the porin genes *ompK35* and *ompK36* (possibly associated with carbapenem resistance, Martínez-Martínez, 2008), the *mgrB* gene, the *pmrA/pmrB* genes, *phoQ/phoP* genes, and the *crrB* gene (possibly associated with colistin resistance, Olaitan et al., 2014b; Wright et al., 2015; Cheng et al., 2016). The mutations observed for the colistin and carbapenem resistant isolates were deduced from NGS data of assembled contigs and confirmed by PCR and subsequent Sanger sequencing (primer sequences: Table S3). Determination of capsular type for *Klebsiella* strains was conducted by *wzi* gene sequencing extracted from contigs derived from *de novo* assembly (Brisse et al., 2013).

### Genetic environment of *bla*_CTX−M−15_

In order to investigate the genetic environment of *bla*_CTX−M−15_, all *bla*_CTX−M−15_-containing contigs derived from *de novo* assembly were aligned (Geneious). Transposases were identified using IS-Finder (https://www-is.biotoul.fr/; Siguier et al., 2006). The transposase upstream of *bla*_CTX−M−15_ in cluster 2 isolates was not completely reconstructed by *de novo* assembly, but by PCR amplification and subsequent Sanger-sequencing (using primers: CTX-M-15-R3: GGTATTGCCTTTCATCCATGTCACCAGC and ST405-R3: CCTTACATTTCAAAAACTCTGCTTACCAGG).

### Pulsed-field gel-electrophoresis (PFGE)

PFGE was performed by the National Reference Laboratory for multidrug-resistant gram-negative bacteria as previously described with some modifications (Ribot et al., 2006). Briefly, genomic DNA was digested with *Xba*I, the initial switch time was 5 s, the final switch time was 50 s, the run time was 20 h, the voltage was 6 V/cm and the temperature was 14°C. Results were interpreted according to the known criteria (Tenover et al., 1995). After treatment with S1 nuclease, resulting DNA fragment pattern were analyzed to predict the size and content of plasmids in the *K. pneumoniae* strains (Barton et al., 1995).

## Results and discussion

### Setting

**Figure 1:**
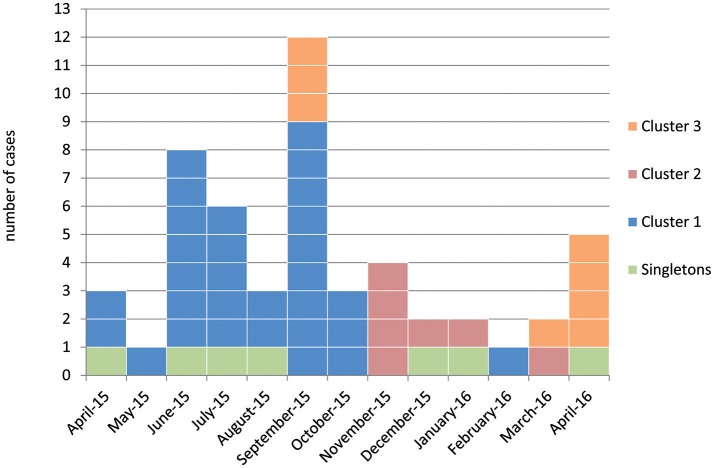
Isolation date of samples received from patients that were tested positive for ESBL-producing *Klebsiella pneumoniae* (*n* = 52) during the outbreak. Colors represent the three outbreak strain clusters and singletons, respectively.

During April and May 2015 ESBL-producing *K. pneumoniae* were detected in nine different patients who just came from the intensive care unit of the neurorehabilitation clinic with overall 202 beds. Taking an average number of three ESBL-*K. pneumoniae* isolates per month in 2014 into account, the infection control specialists suspected an outbreak and an outbreak investigation was started. ESBL-producing *K. pneumoniae* isolates have been detected in 52 patients of whom 20 developed a clinical infection. All ESBL-producing *Klebsiella* isolates collected during the time of the outbreak contained CTX-M-15. Since April 2015, at least one case of each outbreak strain was present on five affected wards (Figure 1). No ESBL-producing *K. pneumoniae* were detected in the environment. Systematic case search with microbiological screening for colonized patients and cohorting of all carriers of ESBL-producing *K. pneumoniae* led to cessation of transmission. After the discharge of the last involved patients in May 2016, no new cases were registered.

### Phylogenetic analysis

**Figure 2:**
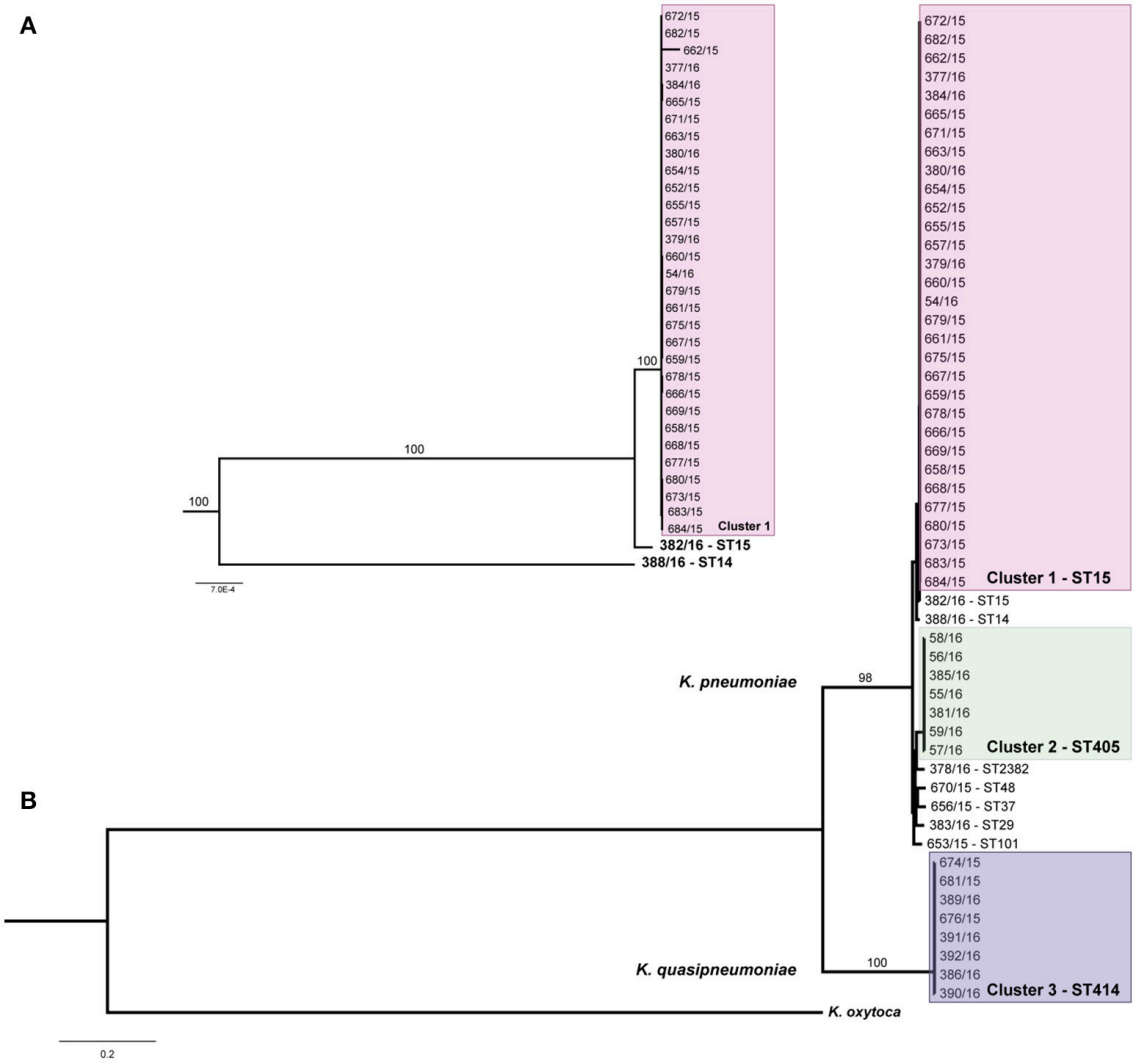
Phylogenetic relationship of all sequenced isolates. **(B)** The maximum likelihood tree was calculated based on 468,250 SNPs filtered after mapping to the reference genome. *K. oxytoca* KONIH1 was included to root the tree. Values on branches display support values (100 bootstraps). The three outbreak clusters are depicted by colored boxes. **(A)** Detailed view including support values of the cluster 1 subtree and the two ST14 and ST15 single isolates. Isolates 680/15 and 684/15 originated from the same patient.

Two different strategies were used to analyse the phylogenetic relations between all 53 *Klebsiella* isolates. First, we applied a reference-based approach by mapping sequence reads to a common reference sequence. As reference we selected *K. pneumoniae* PMK-1 (NZ_CP008929) using *refRank* (see section Materials and Methods for detailed information on reference selection; Figure S1). Second, WGS data of the single isolates were *de novo* assembled and compared using a MCG approach. Subsequent phylogenetic analyses revealed three clonal clusters with 46 isolates and seven unrelated isolates, in total (Figure 2A, Figure S2). Importantly, both the reference-based and -free approach assigned the isolates to the same clusters. Clustering has been also confirmed by PFGE analysis (Table 1).

The major cluster (cluster 1) was formed by 31 isolates belonging to ST15, cluster 2 consisted of seven isolates belonging to ST405 and cluster 3 was formed by eight isolates belonging to ST414. The latter were identified as *K. quasipneumoniae subspecies similipneumoniae* due to the presence of the chromosomal β-lactamase OKP-B (Fevre et al., 2005; Brisse et al., 2014). The classification as *K. quasipneumoniae* was confirmed by *in silico* analysis of the *gyrA* and *parC* genes (Brisse and Verhoef, 2001; Brisse et al., 2004; data not shown). In line with this finding, all ST414 isolates clustered separately. Among the seven non-clustered isolates, one isolate harbored a novel *pgi* allele and was therefore assigned to the novel sequence type ST2382 (http://bigsdb.pasteur.fr/). The other six singletons were belonging to ST15 (different from the outbreak clone), ST14, ST101, ST48, ST29, and ST37.

Cluster 1 *K. pneumoniae* isolates (*n* = 31) belonged to ST15 and were detected nearly during the whole outbreak period (from April 2015 to February 2016). In addition, cluster 1 isolates were received mainly from infections whereas cluster 2 and 3 isolates were almost exclusively collected from rectal swabs. ST15 represents a widely spread international lineage commonly associated with ESBL and carbapenemase genes (Damjanova et al., 2008; Lee et al., 2011; Breurec et al., 2013; Rodrigues et al., 2014). Correspondingly, there are several reports on outbreaks caused by ST15 isolates (Novais et al., 2012; Stoesser et al., 2014; Chung The et al., 2015; Zhou et al., 2016). As depicted in Figure 2B, there was a single isolate also being ST15 but not belonging to the outbreak cluster. While the cluster isolates harbored the capsular gene *wzi*93 (associated K type K60), the single isolate harbored *wzi*24 (associated K type K24; Brisse et al., 2013). These two types represent the two clades previously observed within the ST15 population (Bruchmann et al., 2015; Zhou et al., 2016).

Cluster 2 *K. pneumoniae* isolates (*n* = 7) belonged to ST405 and were detected from November 2015 till March 2016. ST405 also represents an internationally distributed sequence type. In the last years, carbapenemase-producing ST405 have been reported several times in various countries in Europe (e.g., in Spain, France, Italy, and Belgium) and in Yemen (Glupczynski et al., 2012; Gharout-Sait et al., 2014; Liapis et al., 2014; Del Franco et al., 2015; Palacios-Baena et al., 2016; Ruiz-Garbajosa et al., 2016). However, reports on ESBL-producing ST405 are rare; to date there is only one further report on a CTX-M-15-producing ST405 *K. pneumoniae* strain that has caused an outbreak in Spain (Machuca et al., 2016).

Cluster 3 isolates (*n* = 8) belonged to ST414, a member of the species *K. quasipneumoniae*, and occurred in September 2015 (*n* = 2) and from March till April 2016 (*n* = 5) in the clinic. Conspicuously, the occurrence of clinical multidrug resistant *K. quasipneumoniae* is far less frequently reported than the occurrence of *K. pneumoniae*. To the best of our knowledge no cases of *K. quasipneumoniae* outbreaks or clustering have been published so far. According to previous studies, *K. quasipneumoniae* accounts for less than 10 % of the clinical *K. pneumoniae sensu lato* population (Brisse et al., 2004; Maatallah et al., 2014), and hence their potential to cause outbreaks might be proportionally smaller compared to *K. pneumoniae*. However, during the last 2 years there were several announcements of whole genome sequenced clinical *K. quasipneumoniae* isolates and case reports that depict the potential of *K. quasipneumoniae* to cause severe infections (Arena et al., 2015; Breurec et al., 2016; Elliott et al., 2016; Garza-Ramos et al., 2016; Ozer et al., 2016). Underreporting might also be a relevant point related to *K. quasipneumoniae* outbreaks since routine diagnostic methods for species identification such as MALDI-TOF MS provide no reliable differentiation between *K. pneumoniae, K. quasipneumoniae*, and *K. variicola* (Long et al., 2017). Thus, identification of these species requires genotyping (Brisse et al., 2004; Alves et al., 2006; Garza-Ramos et al., 2015) and with regard to the here reported outbreak, the presence of *K. quasipneumoniae* isolates would not have been detected without whole genome-sequencing.

### Antibiotic resistances

Isolates of all three clusters encoded for the ESBL gene *bla*_CTX−M−15_. Further, the three β-lactamase genes *bla*_SHV−28_, *bla*_TEM−1_, and *bla*_OXA−534_, a new allele variant (NCBI accession number KX714285) were found in 26 of 31 isolates belonging to cluster 1 (Table 1). OXA-534 belongs to the OXA-1 group and thus is most likely a narrow spectrum β-lactamase. Cluster 1 isolates displayed resistance to piperacillin, cefotaxime, ceftazidime, cefepime, aztreonam, tobramycin, ciprofloxacin, moxifloxacin, and trimethoprim-sulfamethoxazole, but remained susceptible to imipenem, meropenem, and colistin. Exceptions were two cluster 1 isolates. One isolate (675/125) was resistant to colistin (Vitek 2 MIC >8 mg/L, Etest MIC 16-24 mg/L), although there was no evidence of colistin treatment of the respective patient. Since colistin susceptibility testing using Vitek2 or Etest might be less sensitive, we also performed broth microdilution for a subset of 12 isolates representing all MLST types of CTX-M-15 producing *K. pneumoniae* (Table S1). Only a single isolate (675/15) was colistin-resistant whereas all the other 11 isolates were colistin-susceptible (MICs between 0.125 and 0.5 mg/L). The recently described plasmid-encoded colistin resistance genes *mcr-1-5* (Falgenhauer et al., 2016; Liu et al., 2016; Xavier et al., 2016; Borowiak et al., 2017) could not be detected in this isolate using ResFinder (version 3.0). The *crrB* gene was absent in all cluster 1 strains and thus, alterations in this gene were omitted as a possible source of colistin resistance in our case. Further, there were no alterations in the *pmrB* and the *phoP* genes in this isolate compared to the colistin-susceptible cluster 1 strains. The *mgrB* gene, however, showed a premature stop codon resulting in a putatively truncated protein of 29 amino acids. The same truncation of MgrB was previously detected in three colistin resistant *K. pneumoniae* isolates described in two studies and was reported as a probable source of the colistin resistance (Olaitan et al., 2014a; Poirel et al., 2015). Another isolate of cluster 1 (684/15) showed a non-susceptibility to carbapenem antibiotics (Etest: MIC ertapenem >32 mg/L; MIC meropenem 8 mg/L; MIC imipenem 3 mg/L). This isolate originated from a patient that was treated with meropenem (3 × 2 gram, 8 days, intravenously) after being tested positive for a cluster 1 strain isolate (680/15). Five days after the end of this treatment (= 13 days after the first meropenem dose), the carbapenem-resistant isolate was detected. No carbapenemase genes were detected in the WGS data using ResFinder. However, analysis of the porin genes *ompK35* and *ompK36* exhibited alterations compared to the initial isolate (680/15). The *ompK35* gene was disrupted by insertion of a transposase gene (IS*Ecp1*) after 80 nucleotides, while a single nucleotide deletion (C194-) in the *ompK36* gene resulting in a frameshift and thus a preliminary stop codon at amino acid position 71. Porin loss after meropenem treatment was previously observed in *Klebsiella* isolates belonging to an outbreak in Spain in 2008. After 13 days of treatment, a carbapenem-resistant isolate exhibiting a point mutation resulting in a premature stop codon in the *ompK36* gene was observed (López-Camacho et al., 2014). In our study, all sequenced isolates were checked for alterations in their *ompK* genes. There was no additional isolate with alterations in both the *ompK35* and the *ompK36* genes, but three isolates revealed one single mutated gene each (Table 1). The detected mutations did not influence the carbapenem resistance phenotype or resulted only in slightly increased carbapenem MIC values, respectively (Table S1). This is in concordance with the assumption that increased resistance requires loss of both porins OmpK35 and OmpK36 (Martínez-Martínez, 2008).

### Plasmid content and the genetic environment of *bla*_CTX−M−15_

*De novo* assemblies of Illumina raw reads revealed presence of three small plasmids in all but three cluster 1 isolates. Interestingly, two of these small plasmids were virtually identical to published plasmids. The 3,223 kb-plasmid shared 3,222/3,223 bp with the *Citrobacter freundii*-plasmid *pCAV1321-3223* which encodes the multidrug transporter EmrE (Genbank accession: CP011604). This plasmid seems to be very stable and widespread since the isolate which contained the published plasmid had been collected in Virginia/USA in 2010 (Sheppard et al., 2016), and more plasmids of the same size and with a maximum difference of three nucleotides have been reported from various genera, including *Serratia* (CP011637), *Salmonella* (CP016867), *Enterobacter* (KU302804, CP011569, CP011658), and *Klebsiella* (CP003994, CP01299, CP014305). The 3.559 kb-plasmid shared 3,558/3,559 bp with the *K. pneumoniae*-plasmid pKp_Goe_917-7 (CP018446). The isolate that contained the published plasmid originates, like the isolates of this study, from a German hospital but was collected about 2 years earlier, in 2013. As the annotated genes encode either proteins involved in plasmid replication (replication initiation protein) or hypothetical proteins, conclusions on function and the potential benefit of carrying this plasmid require further analyses. In order to determine the content and the size of plasmids >30 kb, S1 nuclease digestion and subsequent PFGE analysis were exemplarily performed for three cluster 1 isolates. All three isolates revealed presence of plasmids which, however, differed in their content (data not shown). Isolate 652/15 contained one plasmid (approximately 200 kb), isolate 662/15 contained two plasmids (approximately 70 and 200 kb), and isolate 666/15 harbored one plasmid (approximately 230 kb). Thus, there was no evidence for an identical (*bla*_CTX-*M*-15_-carrying) plasmid harbored by all cluster 1 isolates. Although *bla*_CTX-*M*-15_ is primarily found on plasmids, there are also reports of chromosomal localizations (Coelho et al., 2010; Mshana et al., 2015). To investigate the localization of *bla*_CTX-*M*-15_ in the outbreak isolates, the genetic environment of the ESBL gene was compared (Figure S3).

All isolates belonging to cluster 1 and cluster 3 showed an IS*Ecp1* transposase gene (identical to accession AJ242809) upstream of *bla*_CTX-*M*-15_. In contrast, all cluster 2 isolates revealed presence of an IS*15DIV* transposase gene (identical to X13616) upstream of *bla*_CTX-*M*-15_. In most cases the assembled contigs were too small to resolve whether the *bla*_CTX-*M*-15_ gene was located on a plasmid or on the chromosome. However, for 11 isolates of cluster 1 *bla*_CTX-*M*-15_ was found on a contig that also contained the chromosomal encoded *dnaA* gene, arguing for a chromosomal integration. In contrast, in one of the first sampled isolates (652/15) the *bla*_CTX-*M*-15_ gene was located on the same contig as the plasmid encoded replication initiation gene *repB* (*IncR1*; Figure S3). The possible existence of both localizations in cluster 1 isolates suggests either a mobilization of the *bla*_CTX-*M*-15_ during the outbreak or the simultaneous occurrence of both, a chromosomal copy and a plasmid encoded copy of *bla*_CTX-*M*-15_, as recently described for *K. pneumoniae* (Hudson et al., 2014; Zhou et al., 2015).

### A selective PCR set successfully identifies isolates belonging to cluster 1

**Figure 3:**
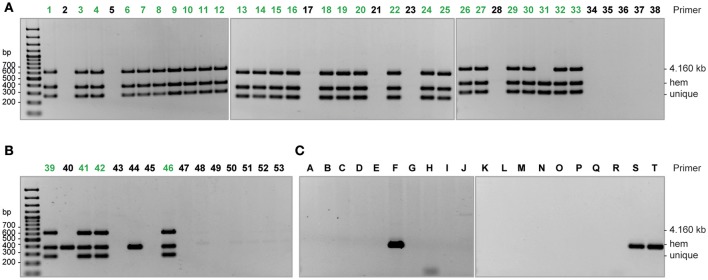
Gel electrophoresis of the cluster 1 specific PCR products (inverted image). PCR products (3 μl) were separated on 1.4% agarose gels. Isolates belonging to the major outbreak strain (cluster 1) are highlighted in green. **(A)** Clinical isolates from the neurorehabilitation clinic that were used to setup the PCR. **(B)** Further clinical isolates that occurred from June 2015 till April 2016 in the neurorehabilitation clinic, and were PCR-typed prior WGS. **(C)** Ten epidemiologically independent ESBL-producing (A–J) and carbapenemase-producing (K–T) *K. pneumoniae* isolates, respectively (see Table S3), used to check the specificity of the PCR. Interpretation: all three bands present: isolate belongs to cluster 1 (“outbreak strain”); all bands present but PCR product of 4,160 kb missing: isolate probably belongs to cluster 1; no PCR product or only amplification of the *hem* product: isolate does not belong to cluster 1.

Since cluster 1 represented the dominant clone during almost the whole outbreak period, an introduction by re-admittance of colonized patients is imaginable. Therefore, a specific multiplex PCR was established allowing rapid detection of emerging cluster 1 isolates to support the hospital laboratory routine (Figure 3). Based on whole genome data of the primarily sequenced 32 isolates, whereof 27 were found to belong to cluster 1, three primer pairs were designed (hem-F/-R, unique-F/-R, and 4.160 kb-F/-R, see section Materials and Methods and Table 2). Isolates revealing presence of all three PCR products were considered to belong to the outbreak cluster/clone (Figure 3A). Isolates with presence of both the *hem* and the *unique* PCR product were considered as probable outbreak isolates since the third primer pair amplifies a plasmid encoded gene and, thus, the absence of the product might be a result of plasmid loss or structural alterations. The latter was the case for cluster 1 isolate 684/15. The PCR succeeded in classifying all further isolates, i.e., the PCR results corresponded with the results of the subsequent whole-genome-analysis (Figure 3B). In order to provide proof of the specificity of the multiplex PCR, 20 unrelated *K. pneumoniae* isolates (Table S2) were tested and results are given in Figure 3C. None of the isolates tested revealed presence of all three bands. The three ST15 isolates showed amplification of the *hem* product, but the absence of the other two targets allowed to discriminate between unrelated ST15 isolates and cluster 1 isolates. We therefore conclude that the designed triplex PCR is a reliable tool for the identification of cluster 1 isolates and suggest for similar situations a combination of two to three discriminating targets for increasing the specificity of a corresponding diagnostic or screening test. Furthermore, the amplification of the *hem* product in all tested ST15 isolates and the absence in all other isolates supports the idea of hem-F/-R as an ST15-specific primer pair. The report of a WGS-based analysis of a *K. pneumoniae* outbreak in a Dutch hospital was published during the outbreak study reported here. The Dutch study included reconstruction of transmission routes and identification of virulence and antibiotic resistance genes, but whole genome sequence data was also used to develop an outbreak-specific triplex PCR (Zhou et al., 2016). Despite differences in the approach applied for the PCR set-up, both studies illustrate the potential of WGS to identify signature regions in outbreak strains.

## Conclusions

WGS-based analysis allowed the successful elucidation of a one-year outbreak with CTX-M-15 producing *K. pneumoniae* in a neurorehabilitation center. Using NGS data and two independent bioinformatics' strategies, we were capable of dividing the scenario into three different outbreak clusters of CTX-M-15 producing *Klebsiella* strain types including one cluster caused by isolates of *K*. *quasipneumoniae*. We performed comprehensive database searches and genomic comparisons to finally deduce a cluster-specific triplex PCR for the main outbreak strain type which (i) was further validated on a valuable set of test and wildtype strains and (ii) allowed a real-time allocation of new *Klebsiella* cases to outbreak and non-outbreak isolates. We did not find experimental proof for a horizontally acquired CTX-M-15 element among strains of the three different WGS clusters considering the different genetic environments as deduced from NGS data. In addition, WGS analysis allowed predicting important antibiotic resistance phenotypes for colistin and carbapenem non-susceptibilities.

## Additional requirements

Sequence data were submitted to the European Nucleotide Archive (http://www.ebi.ac.uk/ena) and are available under study accession number PRJEB18059.

## Ethics statement

No informed consent or ethical approval was required since all isolates were generated and analyzed as part of microbiological diagnostics (therapeutic purposes) and/or infection prevention and control requirements and measures. The outbreak investigation was conducted in accordance with article 25, section 1 of the German Infection Protection Act of 2001.

## Author contributions

LB, SF, YP, SG, HR, and GW: designed the experiments. LB, SF, YP, and TS: performed the experiments. LB, SF, YP, TS, GK, SH, TE, MK, and SG: collected and analyzed the data. LB, MS, TS, and SF: performed bioinformatic analysis. MS and SF: designed and created the Python applications. TE, GK, DS, MF, EZ, CD, M-LT, HR, and SH: were part of the outbreak team, conducted the on-site investigations, implemented control measures, and systematically collected the samples. LB, SF, YP, and GW: wrote the manuscript. All authors reviewed the manuscript.

### Conflict of interest statement

The authors declare that the research was conducted in the absence of any commercial or financial relationships that could be construed as a potential conflict of interest.
