# Supplementary material for: Whole Genome Sequence Analysis of CTX-M-15 Producing Klebsiella Isolates Allowed Dissecting a Polyclonal Outbreak Scenario
Source: Front Microbiol. 2018 Feb 23;9:322. doi: 10.3389/fmicb.2018.00322 (PMC5829066; doi:10.3389/fmicb.2018.00322)
Supplement: Supplementary file 1 [file Table1.PDF]

Table S1. Phenotypic resistance data of selected *Klebsiella* spp. isolates from the neurohabilitation centre.

	Vitek 2 (AST-N248)																	Etest				BMD
**ID<br>MLST<br>(WGS Cluster)**	**PIP**	**TZP**	**CTX**	**CAZ**	**FEP**	**ATM**	**IPM**	**MEM**	**AMK**	**GEN**	**TOB**	**CIP**	**MXF**	**TGC**	**FOF**	**CST**	**SXT**	**IPM**	**ETP**	**MEM**	**CST**	**CST**
652/15<br>ST15 (1)	>64<br>(R)	32<br>(R)	>32<br>(R)	>32<br>(R)	>32<br>(R)	>32<br>(R)	<=0.25<br>(S)	<=0.25<br>(S)	<=2<br>(S)	<=1<br>(S)	>8<br>(R)	>2<br>(R)	>4<br>(R)	<=0.5<br>(S)	<=16<br>(S)	<=0.5<br>(S)	>160<br>(R)	-	-	-	-	1<br>(S)
662/15<br>ST15 (1)	>64<br>(R)	16<br>(I)	>32<br>(R)	>32<br>(R)	>32<br>(R)	>32<br>(R)	<=0.25<br>(S)	<=0.25<br>(S)	4<br>(S)	<=1<br>(S)	>8<br>(R)	>2<br>(R)	>4<br>(R)	<=0.5<br>(S)	<=16<br>(S)	<=0.5<br>(S)	>160<br>(R)	-	-	-	-	1<br>(S)
666/15<br>ST15 (1)	>64<br>(R)	8<br>(S)	>32<br>(R)	16<br>(R)	>32<br>(R)	>32<br>(R)	<=0.25<br>(S)	<=0.25<br>(S)	4<br>(S)	<=1<br>(S)	>8<br>(R)	>2<br>(R)	>4<br>(R)	<=0.5<br>(S)	<=16<br>(S)	<=0.5<br>(S)	40<br>(R)	-	-	-	-	0,5<br>(S)
680/15<br>ST15 (1)	>64<br>(R)	32<br>(R)	>32<br>(R)	>32<br>(R)	>32<br>(R)	>32<br>(R)	<=0.25<br>(S)	<=0.25<br>(S)	<=2<br>(S)	<=1<br>(S)	8<br>(R)	>2<br>(R)	>4<br>(R)	1<br>(S)	128<br>(R)	<=0.5<br>(S)	>160<br>(R)	0,19<br>(S)	-	0,064<br>(S)	-	0,25<br>(S)
684/15<br>ST15 (1)	>64<br>(R)	>64<br>(R)	>32<br>(R)	>32<br>(R)	>32<br>(R)	>32<br>(R)	2<br>(S)	8<br>(I)	<=2<br>(S)	<=1<br>(S)	<=1<br>(S)	>2<br>(R)	>4<br>(R)	<=0.5<br>(S)	>128<br>(R)	<=0.5<br>(S)	<=20<br>(R)	3<br>(I)	>32<br>(R)	4-6<br>(I)	-	0,25<br>(S)
675/15<br>ST15 (1)	>64<br>(R)	32<br>(R)	>32<br>(R)	>32<br>(R)	>32<br>(R)	>32<br>(R)	<=0.25<br>(S)	<=0.25<br>(S)	8<br>(S)	<=1<br>(S)	>8<br>(R)	>2<br>(R)	>4<br>(R)	2<br>(I)	<=16<br>(S)	>8 (R)	>160<br>(R)	0,125<br>(S)	-	0,047<br>(S)	24<br>(R)	32<br>(R)
59/16<br>ST405 (2)	>64<br>(R)	<=4<br>(S)	>32<br>(R)	16<br>(R)	32<br>(R)	>32<br>(R)	<=0.25<br>(S)	<=0.25<br>(S)	<=2<br>(S)	<=1<br>(S)	<=1<br>(S)	<=0.25<br>(S)	2<br>(R)	<=0.5<br>(S)	<=16<br>(S)	<=0.5<br>(S)	>160<br>(R)	0.125<br>(S)	-	0.032<br>(S)	-	0,125<br>(S)
55/16<br>ST405 (2)	>64<br>(R)	16<br>(I)	>32<br>(R)	>32<br>(R)	>32<br>(R)	>32<br>(R)	<=0.25<br>(S)	<=0.25<br>(S)	<=2<br>(S)	<=1<br>(S)	<=1<br>(S)	0.5<br>(I)	2<br>(R)	1<br>(S)	<=16<br>(S)	<=0.5<br>(S)	>160<br>(R)	0.125<br>(S)	0.094<br>(S)	0.032<br>(S)	-	0,125<br>(S)
674/15<br>ST414 (3)	>64<br>(R)	<=4<br>(S)	>32<br>(R)	8<br>(R)	2<br>(I)	16<br>(R)	<=0.25<br>(S)	<=0.25<br>(S)	<=2<br>(S)	<=1<br>(S)	<=1<br>(S)	1<br>(R)	4<br>(R)	1<br>(S)	32<br>(S)	<=0.5<br>(S)	>160<br>(R)	0,125<br>(S)	-	0,016<br>(S)	-	0,125<br>(S)

	Vitek 2 (AST-N248)																	Etest				BMD
**ID**<br>**MLST**<br>**(WGS Cluster)**	**PIP**	**TZP**	**CTX**	**CAZ**	**FEP**	**ATM**	**IPM**	**MEM**	**AMK**	**GEN**	**TOB**	**CIP**	**MXF**	**TGC**	**FOF**	**CST**	**SXT**	**IPM**	**ETP**	**MEM**	**CST**	**CST**
676/15<br>ST414 (3)	>64<br>(R)	<=4<br>(S)	>32<br>(R)	8<br>(R)	2<br>(I)	16<br>(R)	<=0.25<br>(S)	<=0.25<br>(S)	<=2<br>(S)	<=1<br>(S)	<=1<br>(S)	1<br>(R)	4<br>(R)	1<br>(S)	32<br>(S)	<=0.5<br>(S)	>160<br>(R)	-	-	-	-	0,5<br>(S)
378/16<br>ST2382	>64<br>(R)	8<br>(S)	>32<br>(R)	4<br>(I)	>32<br>(R)	>32<br>(R)	<=0.25<br>(S)	<=0.25<br>(S)	<=2<br>(S)	<=1<br>(S)	<=1<br>(S)	0.5<br>(I)	0.5<br>(S)	<=0.5<br>(S)	<=16<br>(S)	<=0.5<br>(S)	<=20<br>(R)	0.38<br>(S)	0.38<br>(S)	0.38<br>(S)	-	0,125<br>(S)
382/16<br>ST15	>64<br>(R)	16<br>(I)	>32<br>(R)	8<br>(R)	2<br>(I)	16<br>(R)	<=0.25<br>(S)	<=0.25<br>(S)	8<br>(S)	>8<br>(R)	>8<br>(R)	>2<br>(R)	>4<br>(R)	2<br>(I)	<=16<br>(S)	<=0.5<br>(S)	<=20<br>(R)	-	-	-	-	0,5<br>(S)
383/16<br>ST29	>64<br>(R)	16<br>(I)	>32<br>(R)	16<br>(R)	2<br>(I)	16<br>(R)	<=0.25<br>(S)	<=0.25<br>(S)	8<br>(S)	>8<br>(R)	>8<br>(R)	2<br>(R)	2<br>(R)	2<br>(I)	>128<br>(R)	<=0.5<br>(S)	>160<br>(R)	-	-	-	-	0,25<br>(S)
388/16<br>ST14	>64<br>(R)	32<br>(R)	>32<br>(R)	16<br>(R)	4<br>(I)	>32<br>(R)	<=0.25<br>(S)	<=0.25<br>(S)	<=2<br>(S)	<=1<br>(S)	8<br>(R)	>2<br>(R)	>4<br>(R)	>4<br>(R)	<=16<br>(S)	<=0.5<br>(S)	>160<br>(R)	-	-	-	-	0,125<br>(S)
653/15<br>ST101	>64<br>(R)	>64<br>(R)	>32<br>(R)	>32<br>(R)	>32<br>(R)	>32<br>(R)	<=0.25<br>(S)	1<br>(S)	16<br>(I)	>8<br>(R)	>8<br>(R)	>2<br>(R)	>4<br>(R)	2<br>(I)	<=16<br>(S)	<=0.5<br>(S)	>160<br>(R)	0.125<br>(S)	1<br>(I)	3<br>(I)	-	0,125<br>(S)
656/15<br>ST37	>64<br>(R)	16<br>(R)	8<br>(R)	<=1<br>(S)	2<br>(I)	4<br>(I)	<=0.25<br>(S)	<=0.25<br>(S)	<=2<br>(S)	<=1<br>(S)	<=1<br>(S)	>2<br>(R)	>4<br>(R)	4<br>(R)	64<br>(R)	<=0.5<br>(S)	>160<br>(R)	-	-	-	-	0,125<br>(S)
670/15<br>ST48	>64<br>(R)	32<br>(R)	>32<br>(R)	>32<br>(R)	>32<br>(R)	>32<br>(R)	<=0.25<br>(S)	<=0.25<br>(S)	8<br>(S)	>8<br>(R)	>8<br>(R)	>2<br>(R)	>4<br>(R)	1<br>(S)	<=16<br>(S)	<=0.5<br>(S)	>160<br>(R)	-	-	-	-	0,5<br>(S)

MIC values (mg/L) as determined by VITEK 2 AST N248®, Etest®, and broth microdilution (BMD), respectively. The letters in brackets refer to the categorization in susceptible (S), intermediate (I) and resistant (R; shaded in grey) according to the EUCAST breakpoints (version 7.1).

Isolates 652/16, 662/15, 666/15, 680/15 belong to cluster 1 (ST15). Isolate 684/15 belongs to cluster 1 (ST15), was collected from the same patient as 680/15 and has a premature stop codon in *ompK36* and an insertion of a transposase in *ompK35*. Isolate 675/15 belongs to cluster 1 (ST15) and has a premature stop codon in *mgrB*. Isolate 59/16 belongs to cluster 2 (ST405). Isolate 55/16 belongs to cluster 2 (ST405) and has a premature stop codon in *ompK35*. Isolate 674/15 belongs to cluster 3 (ST414). Isolate 653/16 is a single isolate (ST101) and has a premature stop codon in *ompK35*. Isolate 378/16 is a single isolate belonging to ST2382 with a premature stop codon in *ompK36*.

Abbreviations: PIP, piperacillin; TZP, piperacillin-tazobactam; CTX, cefotaxime; CAZ, ceftazidime; FEP, cefepime; ATM, aztreonam; IPM, imipenem; MEM, meropenem; AMK, amikacin; GEN, gentamicin; TOB, tobramycin; CIP, ciprofloxacin; MXF, moxifloxacin; TGC, tigecycline; FOF, fosfomycin; CST, colistin; SXT, trimethoprim-sulfomethoxazole; FOX, cefoxitin; ETP, ertapenem
