# Supplementary material for: Whole Genome Sequence Analysis of CTX-M-15 Producing Klebsiella Isolates Allowed Dissecting a Polyclonal Outbreak Scenario
Source: Front Microbiol. 2018 Feb 23;9:322. doi: 10.3389/fmicb.2018.00322 (PMC5829066; doi:10.3389/fmicb.2018.00322)
Supplement: Supplementary file 2 [file Table2.PDF]

**Table S2. List of epidemiologically unrelated isolates used to check the PCR´s specificity**

| | ID | Isolation date | Federal State | Material | MLST | β-Lactamase gene |
|---|---|---|---|---|---|---|
| A | 1/15 | 2015 | Lower Saxony | bronchial secretion | 14 | *bla*$_{CTX-M-15}$ |
| B | 577/15 | 2015 | Lower Saxony | urine | 1263 | *bla*$_{CTX-M-1}$ |
| C | 80/16 | 2016 | Thuringia | rectal swab | 307 | *bla*$_{CTX-M-15}$ |
| D | 4/16 | 2016 | Hesse | wound swab | 395 | *bla*$_{CTX-M-15}$ |
| E | 19/16 | 2016 | Saxony | stool sample | 1192 | *bla*$_{CTX-M-3}$ |
| F | 429/13 | 2011 | Berlin | rectal swab | 15 | *bla*$_{CTX-M-15}$ |
| G | 446/13 | 2013 | Berlin | rectal swab | 405 | *bla*$_{CTX-M-15}$ |
| H | 441/13 | 2012 | Berlin | rectal swab | 29 | *bla*$_{CTX-M-15}$ |
| I | 421/13 | 2011 | Berlin | wound swab | 1399 | *bla*$_{CTX-M-15}$ |
| J | 233/14 | 2011 | Berlin | wound swab | 716 | *bla*$_{CTX-M-15}$ |
| K | 276/15 | 2011 | Lower Saxony | drainage secretion | 101 | *bla*$_{KPC-2}$ |
| L | 294/15 | 2012 | North Rhine-Westphalia | sputum | 512 | *bla*$_{KPC-3}$ |
| M | 301/15 | 2010 | Hesse | urine | 16 | *bla*$_{OXA-48}$ |
| N | 308/15 | 2011 | Brandenburg | urine | 1399 | *bla*$_{OXA-48}$ |
| O | 316/15 | 2012 | Brandenburg | tracheal secretion | 23 | *bla*$_{OXA-48}$ |
| P | 318/15 | 2012 | Saxony | brochoalveolar lavage | 395 | *bla*$_{OXA-48}$ |
| Q | 336/15 | 2013 | Bavaria | tracheal secretion | 16 | *bla*$_{NDM-1}$ |
| R | 311/15 | 2011 | Rhineland-Palatinate | wound swab | 11 | *bla*$_{OXA-48}$ |
| S | 338/15 | 2014 | North Rhine-Westphalia | anal swab | 15 | *bla*$_{NDM-1}$ |
| T | 331/15 | 2014 | Hamburg | pleural punctate | 15 | *bla*$_{OXA-48}$ |
