# Supplementary material for: Whole Genome Sequence Analysis of CTX-M-15 Producing Klebsiella Isolates Allowed Dissecting a Polyclonal Outbreak Scenario
Source: Front Microbiol. 2018 Feb 23;9:322. doi: 10.3389/fmicb.2018.00322 (PMC5829066; doi:10.3389/fmicb.2018.00322)
Supplement: Supplementary file 3 [file Table3.PDF]

Table S3. Primers used for the analysis of carbapenem and colistin resistant isolates

Primer name	Sequence (5´-3´)	Product size	Source
ompK35-a-F	GCAATATTCTGGCAGTGGTG	533 bp	this study
ompK35-a-R	ACGGTCGTGGTCGTTTTTAC		this study
ompK36-a-F	CTCCTGGTACCGGCTCTG	414 bp	this study
ompK36-a-R	GGACTGCAGGAAGTTGTCAG		this study
mgrB_ext_F	TTAAGAAGGCCGTGCTATCC	253 bp	Cannatelli et al., 2013[a]
mgrB_ext_R	AAGGCGTTCATTCTACCACC		Cannatelli et al., 2013[a]

[a] Cannatelli A *et al. In vivo* emergence of colistin resistance in *Klebsiella pneumoniae* producing KPC-type carbapenemases mediated by insertional inactivation of the PhoQ/PhoP *mgrB* regulator.*Antimicrob Agents Chemother* **57**(11), 5521-5526. doi: 10.1128/AAC.01480-13. Epub 2013 Aug 26. (2013)
