# Supplementary material for: Whole Genome Sequence Analysis of CTX-M-15 Producing Klebsiella Isolates Allowed Dissecting a Polyclonal Outbreak Scenario
Source: Front Microbiol. 2018 Feb 23;9:322. doi: 10.3389/fmicb.2018.00322 (PMC5829066; doi:10.3389/fmicb.2018.00322)
Supplement: Supplementary file 4 [file Image1.PDF]

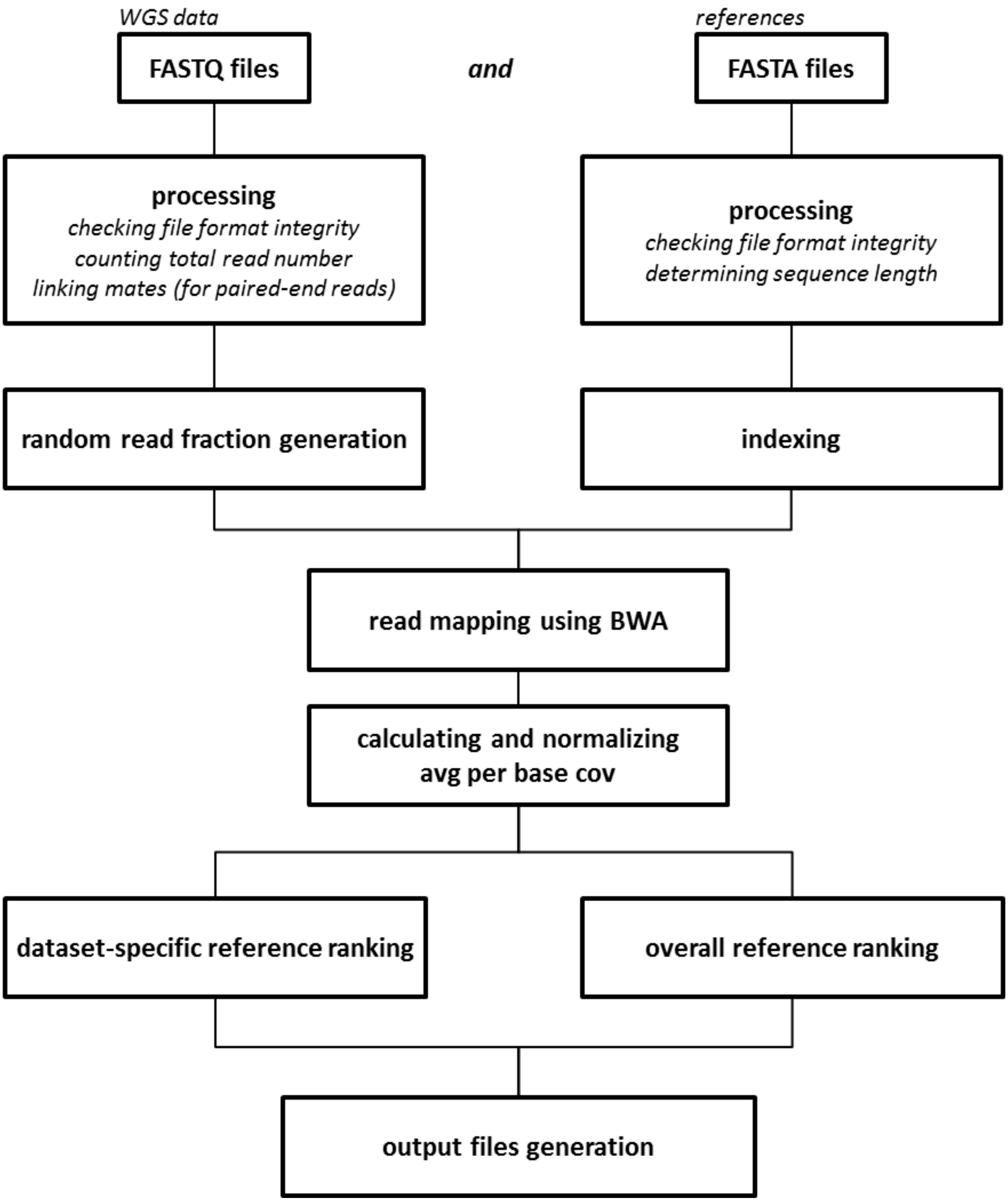

Supplementary Figure S1. Workflow provided by *refRank*. The open-source tool applies BWA to align reads of one or more datasets against a collection of reference sequences. Computational costs can be significantly reduced by the generation of random sub-fractions for each dataset. Based on the resulting alignments the averaged per base coverage (avg per base cov) is calculated for each dataset-reference combination. The avg per base cov is normalized to the number of mapped and unmapped reads and then used to provide dataset-specific and overall reference rankings (for more information see material and methods).
