# Supplementary material for: Whole Genome Sequence Analysis of CTX-M-15 Producing Klebsiella Isolates Allowed Dissecting a Polyclonal Outbreak Scenario
Source: Front Microbiol. 2018 Feb 23;9:322. doi: 10.3389/fmicb.2018.00322 (PMC5829066; doi:10.3389/fmicb.2018.00322)
Supplement: Supplementary file 5 [file Image2.PDF]

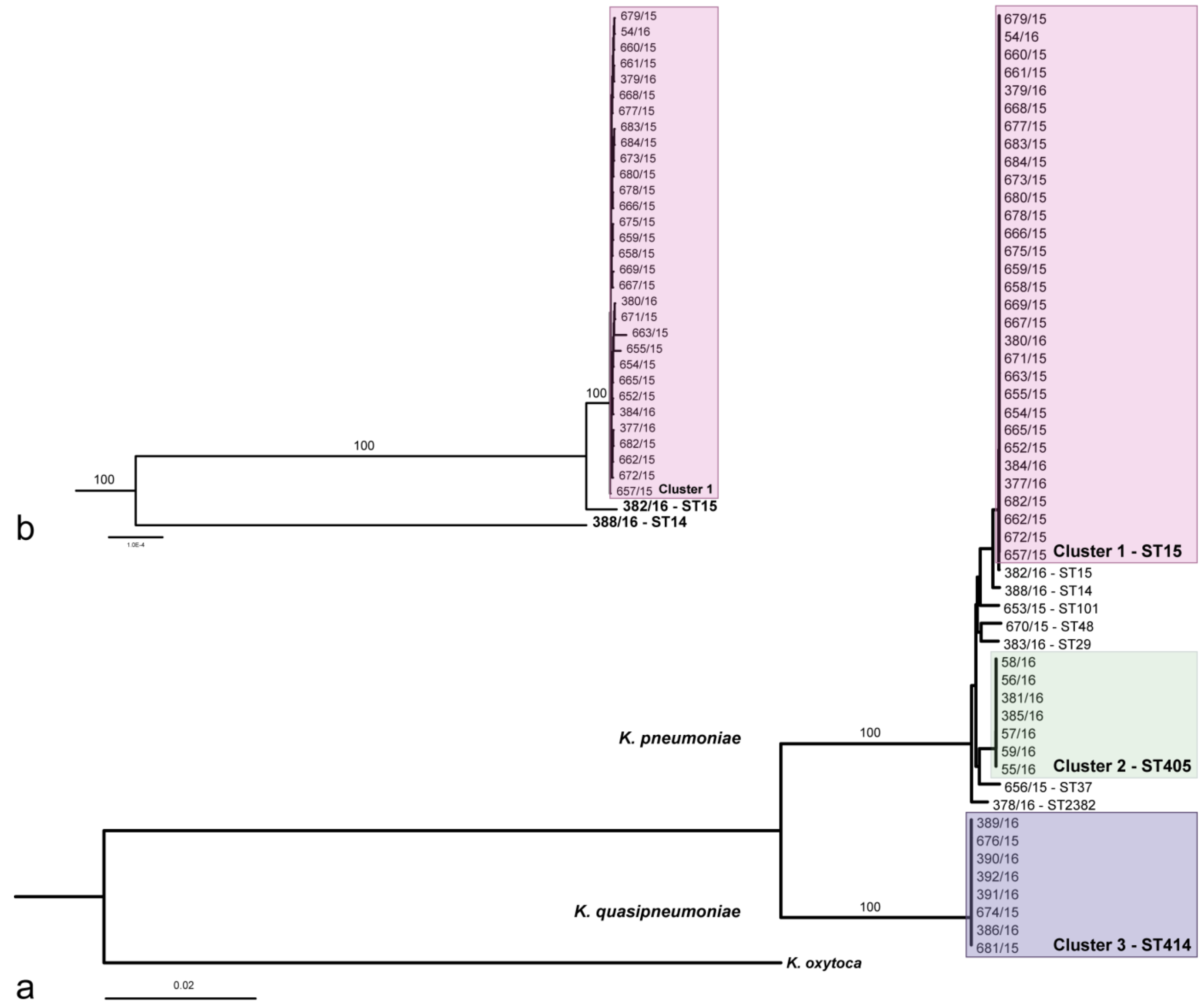

Supplementary Figure S2. Phylogenetic relationship of all sequenced isolates as inferred from the Maximum Common Genome approach. (a) The maximum likelihood tree was calculated based on 1117 orthologous genes. *K. oxytoca* KONIH1 was included to root the tree. Values on branches display support values (100 bootstraps). The three outbreak clusters are depicted by coloured boxes. **(b)** Detailed view including support values of the cluster 1 subtree and the two ST14 and ST15 single isolates. Isolates 680/15 and 684/15 originated from the same patient.
