# Supplementary material for: Whole Genome Sequence Analysis of CTX-M-15 Producing Klebsiella Isolates Allowed Dissecting a Polyclonal Outbreak Scenario
Source: Front Microbiol. 2018 Feb 23;9:322. doi: 10.3389/fmicb.2018.00322 (PMC5829066; doi:10.3389/fmicb.2018.00322)
Supplement: Supplementary file 6 [file Image3.PDF]

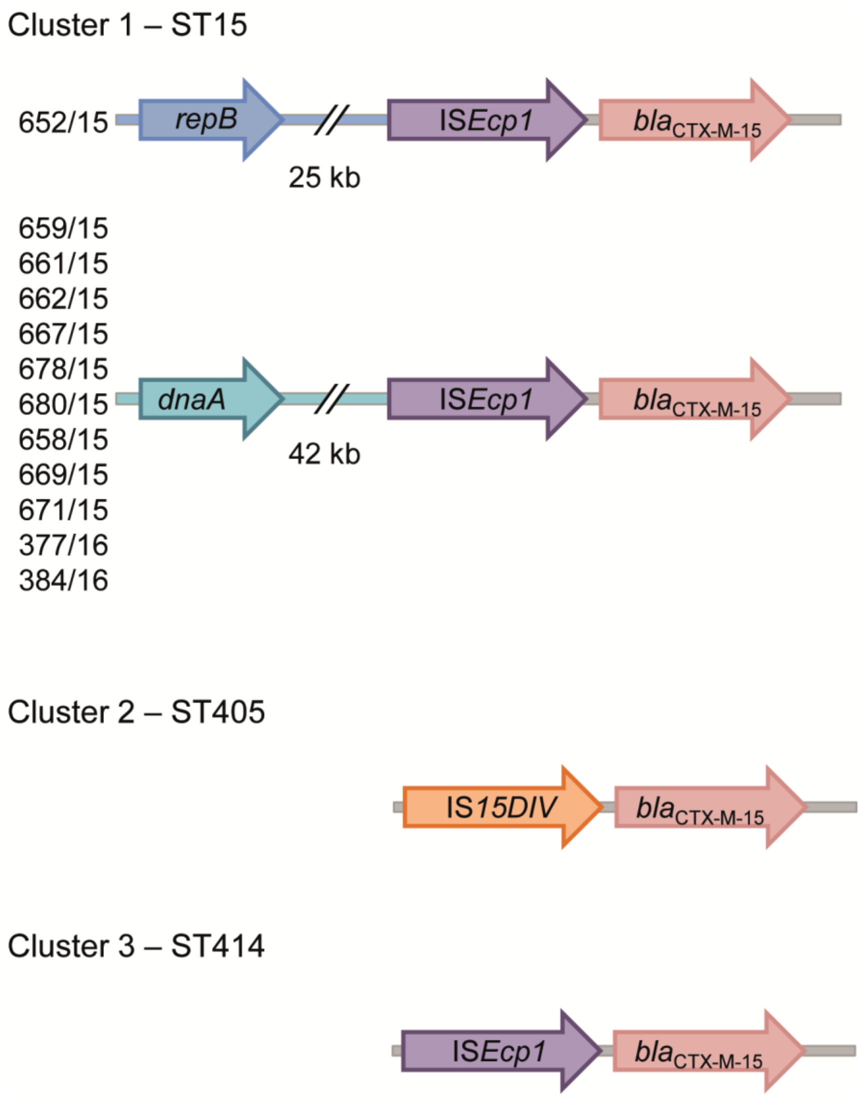

Supplementary Figure S3. Schematic illustration of the genetic environment of *bla*$_{\text{CTX-M-15}}$. Transposase genes found upstream of the *bla*$_{\text{CTX-M-15}}$ gene in contigs derived from *de novo* assembly are shown. Further, genes arguing for a plasmidic (*repB*) or chromosomal (*dnaA*) localisation of the *bla*$_{\text{CTX-M-15}}$ gene, respectively, are displayed.
